# Choosing the most suitable NGS technology to combine with a standardized viral enrichment protocol for obtaining complete avian orthoreovirus genomes from metagenomic samples

**DOI:** 10.3389/fbinf.2025.1498921

**Published:** 2025-02-04

**Authors:** Sonsiray Álvarez-Narváez, Telvin L. Harrell, Islam Nour, Sujit K. Mohanty, Steven J. Conrad

**Affiliations:** ^1^ US National Poultry Research Center, United States Department of Agriculture, Agricultural Research Service, Athens, GA, United States; ^2^ Department of Infectious Diseases, College of Veterinary Medicine, University of Georgia, Athens, GA, United States

**Keywords:** avian orthoreovirus, ARV, whole genome sequencing, WGS, short-read sequencing, long-read sequencing, Oxford Nanopore technologies, ont

## Abstract

Since viruses are obligate intracellular pathogens, sequencing their genomes results in metagenomic data from both the virus and the host. Virology researchers are constantly seeking new, cost-effective strategies and bioinformatic pipelines for the retrieval of complete viral genomes from these metagenomic samples. Avian orthoreoviruses (ARVs) pose a significant and growing threat to the poultry industry and frequently cause economic losses associated with disease in production birds. Currently available commercial vaccines are ineffective against new ARV variants and ARV outbreaks are increasing worldwide, requiring whole genome sequencing (WGS) to characterize strains that evade vaccines. This study compares the effectiveness of long-read and short-read sequencing technologies for obtaining ARV complete genomes. We used eight clinical isolates of ARV, each previously processed using our published viral genome enrichment protocol. Additionally, we evaluate three assembly methods to determine which provided the most complete and reliable whole genomes: *De novo*, reference-guided or hybrid. The results suggest that our ARV genome enrichment protocol caused some fragmentation of the viral cDNA that impacted the length of the long reads (but not the short reads) and, as a result, caused a failure to produce complete genomes via *de novo* assembly. Overall, we observed that regardless of the sequencing technology, the best quality assemblies were generated by mapping quality-trimmed reads to a custom reference genome. The custom reference genomes were in turn constructed with the publicly available ARV genomic segments that shared the highest sequence similarity with the contigs from short-read *de novo* assemblies. Hence, we conclude that short-read sequencing is the most suitable technology to combine with our ARV genome enrichment protocol.

## 1 Introduction

Viruses are obligate intracellular pathogens and when their genomes are sequenced the result is often a metagenome containing both host and viral genomic material. Host genomic contamination impacts both sequencing costs and the quality of viral genome assemblies obtained (Alvarez Narvaez et al., 2023). If no viral genome enrichment is performed prior to sequencing, most sequencing reads (>90%) align with the host genome, leaving only a small fraction of viral reads. Therefore, virology researchers continuously seek cost-effective strategies to obtain viral whole genomes from these metagenomic samples (Goraichuk et al., 2024).

Avian orthoreoviruses (ARVs) are a common threat to poultry producers worldwide. This group of double-stranded RNA (dsRNA) segmented viruses cause a number of health problems in all poultry species, including tenosynovitis, hepatitis, myocarditis, diarrhea, neurological disease, and reduced growth (Benavente and Martinez-Costas, 2007; Egana-Labrin et al., 2019; Lu et al., 2015; Mase et al., 2021). The frequency of ARVs outbreaks has been increasing (Egana-Labrin et al., 2019; Palomino-Tapia et al., 2018; Lu et al., 2015; Liu et al., 2023), even though there are commercial vaccines available. These commercial vaccines are usually made from older viral isolates and are increasingly ineffective against new field isolates (Markis, 2022). Whole genome sequencing (WGS) has become an indispensable tool to characterize ARV strains that escaped vaccine protection (Ayalew et al., 2020; Egana-Labrin et al., 2019). To our knowledge, most of the studies that required the analysis of the complete ARV genome have been performed using short-read Illumina sequencing. Last year, our laboratory developed and published a protocol to enrich ARV genomes from cell cultures prior to Illumina WGS (Alvarez Narvaez et al., 2023). Similarly, our group published the first study that used long-read Oxford Nanopore Technologies (ONT) sequencing for a complete genomic characterization of two field isolates of this viral species (Nour et al., 2023). However, our protocol involved the PCR (polymerase chain reaction) amplification of each of the ten ARV genomic segments individually and their subsequent selection and purification from agarose gels prior genomic library preparation in which each of the ten viral segments was barcoded individually. Although the sequencing results were comparable between the two sequencing technologies (ONT and Illumina), the time and costs associated with the preparation of the ARV genomic material prior ONT sequencing were higher than those required in our optimized enrichment protocol for Illumina sequencing.

In this study, we go one step further in the optimization of ARV WGS and, using the same ARV enrichment method, we compare the performance of long-read ONT sequencing with short-read Illumina sequencing to establish which sequencing technology is the most cost-effective. Furthermore, we compared different bioinformatic pipelines that use *de novo*, reference-guided and hybrid assembly methods to assess the suitability of the different *in silico* analyses to produce complete genomes.

## 2 Materials and methods

### 2.1 Experimental design and viral culture conditions

Eight ARVs were obtained from the Alabama Diagnostic Laboratory System (Auburn, AL, united states of america) and expanded individually in LMH cells (ATCC CRL-2117) at the USDA-ARS US National Poultry Research Center (USDA-ARS, Athens, GA, united states of america). Briefly, LMH cell monolayers at 95% confluency were infected with 20 μL of ARV-infected cell culture supernatant and placed in a cell culture incubator at 38°C, humidified, with 5% CO_2_. After 5 days, the infected LMH cells and supernatant were harvested and centrifuged at 3,000 x g for 10 min at room temperature (RT). The pellet was resuspended in 350 µL of virus dilution buffer (VDB) (James et al., 2016), and subsequently sonicated on ice (3 pulses at 30% amplitude, 10s on and 30s off) using a Branson Digital Sonifier 450 (Branson Ultrasonics Corporation, Brookfield, CT). Sonicated cell pellets containing ARV were subjected to our ARV genome enrichment protocol (https://dx.doi.org/10.17504/protocols.io.14egn38z6l5d/v1 (Alvarez Narvaez et al., 2023)) and the resulting ARV cDNA was split into two aliquots. Half of the sample was used for short-read sequencing library prep and the other half for long-read sequencing.

### 2.2 ARV genome enrichment and genomic libraries preparation

ARV genome enrichment was carried out as previously described by us (https://dx.doi.org/10.17504/protocols.io.14egn38z6l5d/v1). Briefly, an initial virion purification step using Capto Core 700 resin (Cytiva, catalog number GE17-5,481-01) was performed, followed by the depletion of host rRNA (chicken) using custom ssDNA probes (Parris et al., 2022), RNase H (New England Biolabs, catalogue number M0297S) and DNase I (New England Biolabs, catalog number M0303S). Finally, a single primer amplification PCR (R-SPA) was done after cDNA conversion using ARV-specific primers. Short-read genomic libraries were prepared with the Nextera XT DNA Library Preparation Kit (Illumina, catalog number FC-131–1,024) and IDT for Illumina DNA/RNA UD Indexes (Illumina, catalog number 20027,213). The samples were run on an Illumina MiSeq instrument (Illumina) using a MiSeq Reagent Nano Kit v2 500 cycles cartridge (Illumina, catalog number MS-103–1,003). Short-read genomic libraries preparation and sequencing were performed at the USDA-ARS (Athens, GA, United States). Long-read genomic libraries were produced following the Oxford Nanopore Technologies (ONT) Rapid sequencing gDNA barcoding protocol (ONT, catalogue number SQK-RBK110.96) and were run in a GridION platform (ONT). Long-read genomic libraries preparation and sequencing were carried out by Eurofins Genomics LLC (Louisville, KY, United States; www.eurofinsgenomics.com).

### 2.3 Bioinformatic analysis

Illumina (short) raw reads were trimmed, and quality filtered using Trimmomatic (Bolger et al., 2014) with a Phred score threshold greater than 30. The filtered reads were then *de novo* assembled using SPAdes v3.15.3 (Bankevich et al., 2012). ONT (long) raw reads were quality filtered using NanoFilt v2.3.0 (De Coster et al., 2018) and a threshold Q value of 7, and they were subsequently trimmed using Porechop v0.2.4 (Bonenfant et al., 2023) in the GalaxyTrakr online bioinformatic platform (Gangiredla et al., 2021). A *de novo* assembly was performed for the ONT reads using Canu v2.2 (Koren et al., 2017) and five different expected genome sizes: 1kb, 2kb, 3kb, 4kb, 23 kb. Additionally, a second *de novo* assembly of the ONT reads was performed using Flye v2.9.1 (Kolmogorov et al., 2019). The resulting contigs from all *de novo* alignments were mapped against the S1133 reference genome (NCBI accession numbers KF741756 - KF741765) and extracted using the Geneious mapper, configured to the highest sensitivity and set for five iterations in Geneious Prime bioinformatics platform [Geneious Prime 2022.1.1, https://www.geneious.com]. For each ARV isolate, the nucleotide sequence similarity shared by a particular genomic segment obtained with different sequencing technologies and pipelines was determined by performing multiple alignments with Clustal Omega v1.2.3 (Sievers and Higgins, 2014) also in Geneious Prime. Additionally, BLASTn algorithm (Altschul et al., 1990) was used to map all ARV contigs obtained with both sequencing technologies to the NCBI database. The reference-guided assemblies were performed using an in-house pipeline (Supplementary Figure S1) that involved BWA v0.7.17 (Li and Durbin, 2010) or minimap2 v2.28 (Li, 2016), SAMtools v1.16.1 (Li et al., 2009) and BCFtools v1.15.1 (Li, 2011). SAMtools v1.16.1 was also used to estimate the coverage and sequencing depth of the reference-guided assembled genomes. Hybrid genome assemblies were obtained by combining filtered short- and long-reads in Unicycler v 0.4.8.0 (Wick et al., 2017) in GalaxyTrakr online bioinformatic platform. The NCBI accession numbers for all genomes used in this study are included in supplementary materials (Supplementary Table S2). Similarly, the raw reads generated during this project can be found in the NCBI Sequence Read Archive (SRA) under bioproject PRJNA1156059.

### 2.4 Statistical analysis

Multiple paired t-tests were used to assess the significant differences in the genome coverage using the different reference genomes in the reference guided assemblies. The Wilcoxon signed-rank test was used to determine the significant differences in the read and sequencing depth fold change of using the custom genomes compared to the S1133 genome in the reference guided assemblies. A threshold of *p* < 0.05 was used to determine statistical significance. All analyses were performed in statistical software GraphPad Prism 9.3.1 (La Jolla, United States).

## 3 Results

### 3.1 ONT-reads *de novo* alignment did not result in complete genomes

Illumina short-read sequencing produced 936,246 raw reads of which 542,334 passed quality filtering. An average of approximately 92% of filtered reads per isolate (58,967 ± 11,110 reads, mean ± SEM) were found to be ARV-mapping reads (Supplementary Table S3). ONT long-read sequencing resulted in a total of 59,839 raw reads (26,115,194 bp) with a median read length of 348 bp and a median read quality of 11. For every sample, more than 86% of the reads passed the quality filtering and trimming process (6,663 ± 413 reads, mean ± SEM) and an average of 60% of those filtered reads per isolate (3,957 ± 86 reads, mean ± SEM) mapped with the ARV reference genomes (Supplementary Table S4).

**FIGURE 1:**
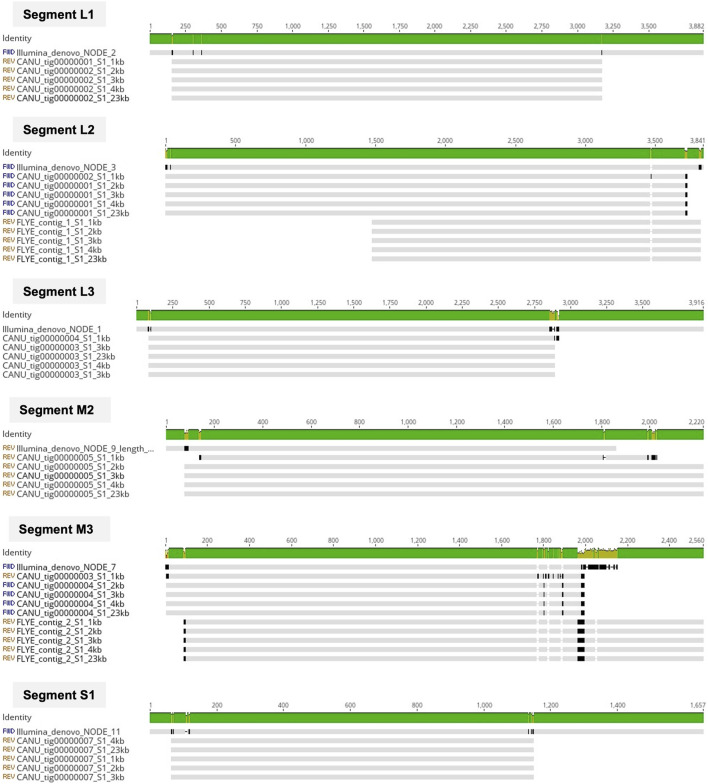
Multiple alignment of common contigs generated by the *de novo* assemblies of Illumina (short) and ONT (long) reads for sample 1. Contigs were identified as segments L1, L2, L3, M2, M3 and S1. The grey horizontal lines represent the contig from the Illumina *de novo* assemblies (Illumina_SPAdes) and the ten *de novo* generated ONT contigs using different expected genome sizes (1kb, 2kb, 3kb, 4kb, 23 kb) and assemblers (Canu and Flye). Vertical black lines indicate disagreement between the nucleotide sequences. The Identity bar summarizes sequence similarity shared among all contigs, ranging from green if a particular nucleotide is shared by all sequences, to red if it is not shared by any. Alignment visualized in Geneious Prime (Geneious Prime 2022.1.1, https://www.geneious.com).

After genome polishing, the number of genomic segments obtained using the short-read sequencing data in a *de novo* assembly ranged between 10 and 19 complete segments per isolate (Table 1), indicating that this method detected more than one ARV strain in some samples (n = 7). Surprisingly, the *de novo* assembly of the ONT long-reads never resulted in complete genomes containing the characteristic 10 segments. In our first attempt we run Canu assembler that uses an overlap-layout-consensus (OLC) algorithm, and five different expected genome sizes based on the ARV genomic segment size range (1kb, 2kb, 3kb, 4 kb) and the size of the viral whole genome (23 kb) to assemble the ONT reads. The resulting assemblies presented between five and nine segments per sample (Table 1) and were identical in length regardless of the expected genome size selected except for when using 1kb, with which we obtained less, and shorter genomic segments compared to the other expected genome sizes. The presence of more than one ARV genome evidenced by the Illumina *de novo* assemblies was detected in three of the eight isolates with the ONT data (samples 2, 3 and 7). Still, seeing that the long-read assemblies produced with the OLC algorithm did not deliver complete genomes, we tried another assembler, Flye that uses a modification of the de-Bruijn-graph (DBG) algorithm instead of OLC. Unfortunately, this assembler produced even less genomic segments than Canu (Table 1), indicating that both OLC and DBG algorithms, designed for long reads, struggle to assemble smaller reads. For each isolate, we assessed the nucleotide sequence similarity of the same genomic segment obtained with the different sequencing technologies and pipelines and we observed that the ONT assemblies (except for when using 1 kb as the expected genome size) and the Illumina assemblies shared >99% sequence similarity independently of the isolate and genomic segment analyzed, (Figure 1).

**TABLE 1:** ARV genomic segments obtained with different *de novo* assembly methods.

Seq ID	Isolate ID	Illumina short reads *de novo*	ONT long reads *de novo* with OLC algorithm	ONT long reads *de novo* with DBG algorithm	Hybrid
S1	1197	19	6	2	19
S2	1148	12	9	1	12
S3	1110	13	6	0	12
S4	1143	10	6	1	10
S5	1072	13	5	1	15
S6	1064	11	8	1	11
S7	1087	13	7	1	15
S8	1088	12	5	4	18

### 3.2 The importance of selecting the right reference genome for reference-guided assemblies

**FIGURE 2:**
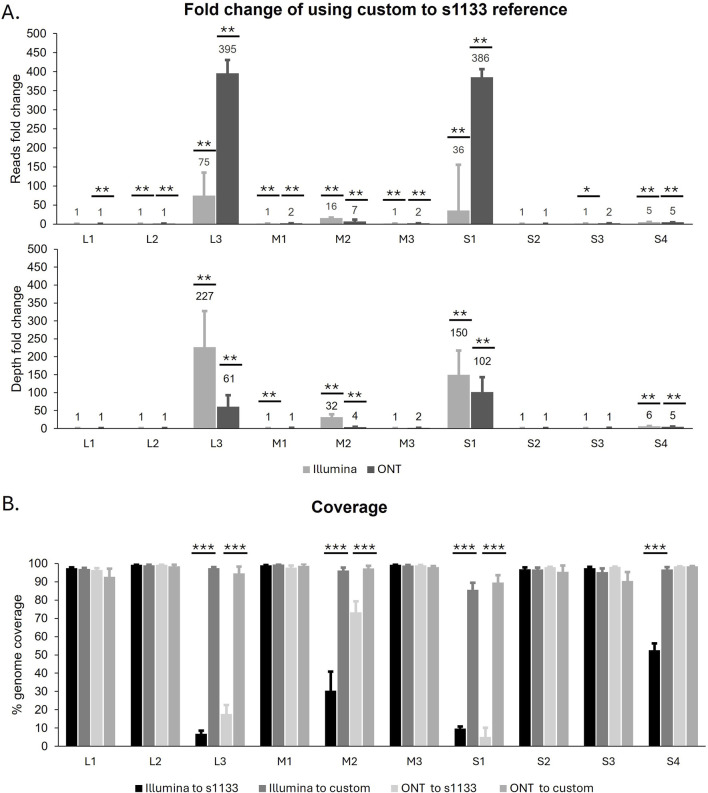
**(A)** Fold change of reads and sequencing depth from raference-based assemblies obtained usign a custom genomes compare to ARV S1133 genome (*Y*-axis), organized by genomic segment (*X*-axis). Bars represent the mean ± SEM of eight samples. **(B)** Percentage of genome coverage obtained with the reference-based assembly and different sequencing data (Illumina or ONT) and ARV reference genomes (S1133 or custom). Bars represent the mean ± SEM and significant differences between groups are denoted with an asterisk (* *p* < 0.05, ** *p* < 0.01, *** *p* < 0.001).

Both the Illumina and the ONT reads were used in reference-guided assemblies using two different types of reference genomes. First, the quality-filtered reads from both technologies were assembled using the well-characterized ARV S1133 genome (NCBI accession number KF741756- KF741765) as a reference. Additionally, all ARV genomic segments obtained with both sequencing technologies during the *de novo* assemblies were mapped to the NCBI database to identify their highest sequence similarity at the nucleotide level. These sequences were used to create eight custom reference genomes, one for each of the new ARV genomes we wished to assemble (Supplementary Table S2). We observed that, regardless of the sequencing method, a significantly higher number of reads mapped to the custom reference genomes compared to the S1133 genome. This in turn translated to a significantly higher genome coverage and sequencing depth for the assemblies produced using the custom genomes as a reference (Table 2). Furthermore, when we look at the guided assemblies for each of the ARV genomic segments individually (Figure 2), the highest differences between using S1133 or the custom genomes as reference are found in segments L3 and S1. On average, the number of Illumina reads that mapped with the custom L3 and S1 segments was 75 and 36 times higher than the number of reads that mapped with the same segment in the S1133 genome respectively. These differences were even greater when using the ONT long reads, for which in many cases no reads were observed to map to the S1133 L3 and S1 segments (Supplementary Table S5). This indicates that the L3 and S1 sequences in the S1133 genome are very different from the ones of the recently sequenced isolates and the assembler does not find enough similarity to map reads to them. Consequently, the coverage and sequencing depth of the L3 and S1 assemblies generated using the custom genomes were also significantly higher (Figure 2). Although less extreme, significant differences were also observed in the M2 and S4 reference-guided assemblies, particularly noticeable for the short reads assembled using the S1133 genome as a reference, which were significantly worse (Figure 2).

**TABLE 2:** Summary of the reference-guided assembly results in terms of number of short (Illumina) and long (ONT) reads that map to the ARV S1133 genome and to the custom genome, average genome coverage and average sequencing depth. A threshold of *p* < 0.05 was used to determine statistical significance.

	Illumina short reads			ONT long reads		
	S1133 ref.	Custom ref.	Significance	S1133 ref.	Custom ref.	Significance
Total mapped reads	364,781	531,648	*p* = 0.0013; t = 5.154, df = 7	16,126	33,347	*p* < 0.0001; t = 10.84, df = 7
Average genome coverage	68.9 ± 12.6	96.4 ± 1.3	*p* < 0.0001; t = 20.87, df = 7	78.3 ± 11.5	95.7 ± 1.0	*p* < 0.0001; t = 10.96, df = 7
Average sequencing depth	293.0 ± 106.5	429.0 ± 79.1	*p* = 0.0108; t = 3.440, df = 7	38.0 ± 7.8	67.1 ± 7.3	*p* = 0.0018; t = 4.853, df = 7

### 3.3 Hybrid assemblies are not superior to refence-guided assemblies at generating ARV complete genomes

**FIGURE 3:**
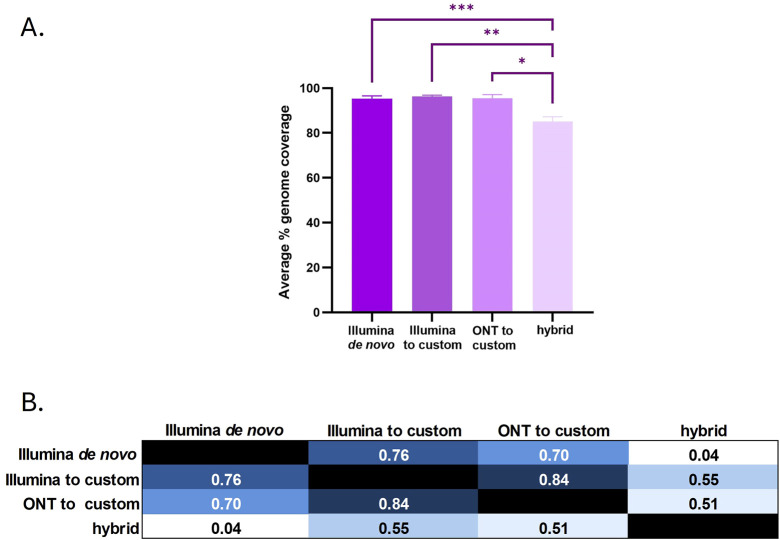
**(A)** Average of percentage genome coverage obtained with the different assembly methods. Bars represent the mean ± SEM and significant differences between groups are denoted with an asterisk (*p < 0.05, **p < 0.01, ***p < 0.001). **(B)** Overall genomic variability shared between the ARV genomes obtained with the different assembly methods. Numbers refer to the percentage of different nucleotides found between groups. The intensity of the color evidences sequences dissimilarity from higher (dark blue) to lower (white).

Finally, we explored the utilization of hybrid assemblies that combine short and long reads to produce complete ARV genomes. As observed in the short-read *de novo* assemblies, the hybrid assemblies could identify the presence of more than one ARV genome in all the samples except for sample 4 (Table 1). In most of the cases, the hybrid assemblies did not produce complete genomic segments, and their length did not exceed what obtained with the *de novo* assembly of short reads. Overall, the longest genome assemblies were obtained mapping the Illumina short-reads to a custom reference genome, followed by reference-guided assemblies of ONT (also to the custom genome), then short-read *de novo* assemblies, and, finally, the hybrid assemblies (Figure 3A). The ONT *de novo* assemblies as well as the reference-guided assemblies using S1133 as the reference genome were not included in this last comparison because of their lower quality that failed to generate at least one version of each of the ARV genomic segments. A closer look at the sequence similarity of the genomic segments obtained with the different assembly methods (short-read *de novo*, reference-guided with custom genomes and hybrid) showed that their minimum nucleotide sequence similarity was >99.1% indicating that the assemblies were very similar, only diverging at a few nucleotide positions (Supplementary Table S6). The two most similar assemblies were the hybrid and the Illumina *de novo* assemblies, with only 0.04% of their nucleotide sequence being different. This indicates that the hybrid assemblies did not improve the results obtained by using the Illumina short reads exclusively. As expected, the number of nucleotide differences increased with the number of extra segments found in the genomes, and more differences were observed at the end of the segments where the sequencing depth was lower. This explains why the hybrid assemblies (which were considerably shorter) shared the least differences with the other assemblies. Surprisingly, the highest number of nucleotide differences between two assemblies were observed when comparing the assemblies generated using the custom genomes as a reference (Figure 3B). We suspect this difference might be due to the different error rates of the two sequencing technologies.

## 4 Discussion

In this study, we advance the optimization of ARV whole-genome sequencing (WGS) by using our previously-published ARV enrichment methods to compare the performance of long-read ONT sequencing with short-read Illumina sequencing, aiming to determine the most cost-effective sequencing workflow. Additionally, we evaluated various bioinformatic pipelines, including *de novo*, reference-guided, and hybrid assemblies, to assess their effectiveness in producing complete genomes through different *in silico* analyses.

The percentage of ARV-mapping reads in this experiment (∼90%) was slightly higher than in our previous experiments in which we obtained ∼70% of short reads mapping to the ARV genome (Alvarez Narvaez et al., 2023; Alvarez Narvaez et al., 2024), and substantially higher than other ARV WGS efforts that did not implement our ARV enrichment protocol (Tang et al., 2022; Egana-Labrin et al., 2019). A similar percentage of ARV reads was observed using long-read sequencing (ONT), evidencing that our ARV enrichment method is suitable to be used with this sequencing technology as well. Surprisingly, the average length of the long reads never exceeded 500 bp for any sample. We suspect that some part of our host rRNA depletion protocol might have impacted the length of ARV RNA segments, resulting in shorter cDNAs and therefore shorter reads. While the RNase H and the DNase I used in this project have been reported by the manufacturer not to digest single- or double-stranded RNA, we cannot discount the possibility that the required manipulation of the RNA during sample processing might have resulted in some RNA shearing. Notably, a previous study that used a random reverse transcription/amplification protocol to amplify viral DNA and RNA, similar to the one we used in this study, also yielded shorter (average < 800bp) ONT reads than was expected (Vigil and Aw, 2023). For these reasons we cannot ignore the possible negative impact of the ARV RNA transcription/amplification steps during sample processing on the resulting ONT read length.

The genomic fragmentation that happens during the viral genome enrichment process did not seem to impact the short-read sequencing outcomes as the resulting genomic fragments were bigger than the length of the reads produced with Illumina. In fact, the results obtained with *de novo* assemblies of the short-read data demonstrate that most of the isolates analyzed included more than one ARV genome. This is not unexpected and is consistent with previous findings from us genome (Alvarez Narvaez et al., 2023; Alvarez Narvaez et al., 2024) and others (Tang et al., 2016; Spackman et al., 2005; Moura-Alvarez et al., 2013; Jindal et al., 2010) evidencing that ARV coinfections are a common event that often goes undetected unless the isolate is subjected to deep sequencing methods. It also shows that researchers wishing to conduct studies on particular strains of ARV must first verify that those strains are pure. The fact that only a few of the 10 genomic segments that compose the ARV genome appeared to be duplicated but not all can be explained in at least three ways: (i) it could be possible that our sequencing depth would not allow us to decipher the complete genome of the ARV in less abundance (Alvarez Narvaez et al., 2024); (ii) another possibility is that the two ARV genomes found infecting a particular bird shared some (but not all) of their genomic segments (Tang et al., 2016); (iii) and finally our observations could be due to a technical error of the assembler. If the genomic sequences of the two coinfecting ARVs share a high sequence similarity, the assembler might have produced a chimeric segment (Arroyo Mühr et al., 2020; Castro et al., 2020). The long-read ONT *de novo* assemblies yielded less and shorter contigs than their short-read Illumina counterparts, indicating that this sequencing strategy combined with our ARV enrichment methods was less suitable for *de novo* genome assemblies. The two most common graph algorithms employed by *de novo* assembly programs are the de-Bruijn-graph (DBG) algorithm mainly used by short-read assemblers such as SPAdes (Bankevich et al., 2012), and the OLC algorithm commonly used by long-read assemblers such as Canu (Li et al., 2011; Koren et al., 2017). We hypothesized that the small size of the ONT reads might be challenging for the OLC software, and we performed a second *de novo* assembly of the ONT data using Flye (Kolmogorov et al., 2019), an assembler for long error-prone reads that uses the DBG algorithm instead. However, Flye performed worse than Canu, providing less and shorter contigs. This suggests that regardless of the algorithm, long-read assemblers have a hard time scaffolding shorter reads.

When a reference genome is available, reference-based assemblies are preferred by many to generate viral whole genomes as they are considered to be more accurate than the *de novo* methods (Fu et al., 2023). Generally, a well characterized and complete reference genome is used in this process. This works well for non-segmented viruses that do not have the opportunity to recombine via reassortment. When two or more segmented RNA viruses (such as ARV) co-infect a host cell, their genomic segments can reassort or “shuffle” leading to the creation of progeny viruses with novel genome combinations (Vijaykrishna et al., 2015). If this progeny’s genome was to be sequenced and assembled using either of the parental strains as reference there will be many reads that will not map to the selected parental reference because they belong to a segment obtained from the other parental virus. Taking into consideration the segmented nature of ARV and its genomic diversity arising from frequent reassortment events (Farkas et al., 2016; Alvarez Narvaez et al., 2024), we explored the utilization of custom reference genomes constructed based on segments of all publicly available ARV genomes. As expected, based on the reasoning above, more reads mapped to the custom genomes than to the reference genome of S1133. This difference was especially evident in the less conserved segments of the genome (Egana-Labrin et al., 2021), such as L3 and S1 carrying genes encoding for the turret Lambda C and the cell attachment protein and major antigen Sigma C respectively (Benavente and Martinez-Costas, 2007), supporting the idea that those particular segments in the tested isolates are genetically very different from the selected reference genome. This significant increase in the number of reads mapping to the custom genomes translated in a higher coverage and sequencing depths in these assemblies compared to the ones generated with S1133 reference genome, demonstrating that the use of custom genomes should be implemented when performing a reference-guided assembly of ARV genomes.

A few years ago, hybrid genome assemblies, which combine the advantages of short- and long-read sequencing technologies, became a very popular strategy for the assembly of long and/or complicated genomes (Di Genova et al., 2021; De Maio et al., 2019). In this assembly method, the long reads (more prone to errors) are used to scaffold the genome and the short reads (with a lower error rate) are used to correct the errors in the preliminary scaffold, leading to, in theory, a more complete and precise genome assembly (Wick et al., 2017). However, we did not observe this with our samples. The hybrid assemblies were the shortest when compared to the Illumina *de novo* assemblies and the reference-based assemblies using the custom genomes. Additionally, the genomes produced were genetically nearly identical (>99%) to the ones observed just using the data from a single sequencing technology. These finding might be explained by the reduced length of the long reads that do not seem to improve the scaffolding process, and by the fact that the ARV genome is small (∼23 kb) and not particularly complex. Both the Illumina, *de novo* and hybrid assemblies detected the presence of more than one ARV in seven of the eight samples, but the ARV genomic segments that did not result in a good depth of coverage appeared incomplete and some segments were missing. ONT sequencing claims to have an average read length of ∼10 kb (Wang et al., 2021), more than twice as long as the longest ARV genomic (the L1 segment is < 4kb (Benavente and Martinez-Costas, 2007)). Hence, under optimal conditions ONT sequencing would produce long enough reads to cover a complete genomic segment, and that would be helpful in the genomic characterization of samples that carry more than one ARV isolate. Future work would include the optimization of our ARV enrichment protocol to provide better genetic materials for ONT sequencing.

In conclusion, the ARV enrichment procedures that we commonly use to increase the proportion of ARV in the metagenomes resulted in fragmentation of the viral genetic material which negatively impacted the length of the ONT sequencing outcome. Therefore, Illumina short-read sequencing is currently the most suitable sequencing technology to be used with our ARV enrichment protocol, and the reference-based assemblies using a custom reference genome the method that provides the most complete genomes.

## Data Availability

The datasets presented in this study can be found in online repositories. The names of the repository/repositories and accession number(s) can be found below: https://www.ncbi.nlm.nih.gov/, bioproject PRJNA1156059.

## References

[B1] Altschul S. F. Gish W. Miller W. Myers E. W. Lipman D. J. (1990). Basic local alignment search tool. J. Mol. Biol. 215, 403–410. 10.1016/s0022-2836(05)80360-2 2231712

[B2] Alvarez Narvaez S. Harrell T. L. Day J. M. Conrad S. J. (2024). Whole genome sequence analysis of Turkey orthoreovirus isolates reveals a strong viral host-specificity and naturally occurring co-infections in commercial turkeys. Virology 600, 110216. 10.1016/j.virol.2024.110216 39293236

[B3] Alvarez Narvaez S. Harrell T. L. Oluwayinka O. Sellers H. S. Khalid Z. Hauck R. (2023). Optimizing the conditions for whole-genome sequencing of avian reoviruses. Viruses 15, 1938. 10.3390/v15091938 37766345 PMC10536876

[B4] Arroyo Mühr L. S. Lagheden C. Hassan S. S. Kleppe S. N. Hultin E. Dillner J. (2020). *De novo* sequence assembly requires bioinformatic checking of chimeric sequences. PLoS One 15, e0237455. 10.1371/journal.pone.0237455 32777809 PMC7417191

[B5] Ayalew L. E. Ahmed K. A. Mekuria Z. H. Lockerbie B. Popowich S. Tikoo S. K. (2020). The dynamics of molecular evolution of emerging avian reoviruses through accumulation of point mutations and genetic re-assortment. Virus Evol. 6, veaa025. 10.1093/ve/veaa025 32411390 PMC7211400

[B6] Bankevich A. Nurk S. Antipov D. Gurevich A. A. Dvorkin M. Kulikov A. S. (2012). SPAdes: a new genome assembly algorithm and its applications to single-cell sequencing. J. Comput. Biol. 19, 455–477. 10.1089/cmb.2012.0021 22506599 PMC3342519

[B7] Benavente J. Martinez-Costas J. (2007). Avian reovirus: structure and biology. Virus Res. 123, 105–119. 10.1016/j.virusres.2006.09.005 17018239

[B8] Bolger A. M. Lohse M. Usadel B. (2014). Trimmomatic: a flexible trimmer for Illumina sequence data. Bioinformatics 30, 2114–2120. 10.1093/bioinformatics/btu170 24695404 PMC4103590

[B9] Bonenfant Q. Noe L. Touzet H. (2023). Porechop_ABI: discovering unknown adapters in Oxford Nanopore Technology sequencing reads for downstream trimming. Bioinform Adv. 3, vbac085. 10.1093/bioadv/vbac085 36698762 PMC9869717

[B10] Castro C. J. Marine R. L. Ramos E. Ng T. F. F. (2020). The effect of variant interference on *de novo* assembly for viral deep sequencing. BMC Genomics 21, 421. 10.1186/s12864-020-06801-w 32571214 PMC7306937

[B11] DE Coster W. D'Hert S. Schultz D. T. Cruts M. VAN Broeckhoven C. (2018). NanoPack: visualizing and processing long-read sequencing data. Bioinformatics 34, 2666–2669. 10.1093/bioinformatics/bty149 29547981 PMC6061794

[B12] DE Maio N. Shaw L. P. Hubbard A. George S. Sanderson N. D. Swann J. (2019). Comparison of long-read sequencing technologies in the hybrid assembly of complex bacterial genomes. Microb. Genom 5, e000294. 10.1099/mgen.0.000294 31483244 PMC6807382

[B13] DI Genova A. Buena-Atienza E. Ossowski S. Sagot M. F. (2021). Efficient hybrid *de novo* assembly of human genomes with WENGAN. Nat. Biotechnol. 39, 422–430. 10.1038/s41587-020-00747-w 33318652 PMC8041623

[B14] Egana-Labrin S. Hauck R. Figueroa A. Stoute S. Shivaprasad H. L. Crispo M. (2019). Genotypic characterization of emerging avian reovirus genetic variants in California. Sci. Rep. 9, 9351. 10.1038/s41598-019-45494-4 31249323 PMC6597705

[B15] Egana-Labrin S. Jerry C. Roh H. J. Da Silva A. P. Corsiglia C. Crossley B. (2021). Avian reoviruses of the same genotype induce different pathology in chickens. Avian Dis. 65, 530–540. 10.1637/0005-2086-65.4.530 35068095

[B16] Farkas S. L. Marton S. Dandar E. Kugler R. Gal B. Jakab F. (2016). Lineage diversification, homo- and heterologous reassortment and recombination shape the evolution of chicken orthoreoviruses. Sci. Rep. 6, 36960. 10.1038/srep36960 27830770 PMC5103266

[B17] Fu P. Wu Y. Zhang Z. Qiu Y. Wang Y. Peng Y. (2023). VIGA: a one-stop tool for eukaryotic virus identification and genome assembly from next-generation-sequencing data. Brief. Bioinform 25, bbad444. 10.1093/bib/bbad444 38048079 PMC10753531

[B18] Gangiredla J. Rand H. Benisatto D. Payne J. Strittmatter C. Sanders J. (2021). GalaxyTrakr: a distributed analysis tool for public health whole genome sequence data accessible to non-bioinformaticians. BMC Genomics 22, 114. 10.1186/s12864-021-07405-8 33568057 PMC7877046

[B19] Goraichuk I. V. Harden M. Spackman E. Suarez D. L. (2024). The 28S rRNA RT-qPCR assay for host depletion evaluation to enhance avian virus detection in Illumina and Nanopore sequencing. Front. Microbiol. 15, 1328987. 10.3389/fmicb.2024.1328987 38351914 PMC10864109

[B20] James K. T. Cooney B. Agopsowicz K. Trevors M. A. Mohamed A. Stoltz D. (2016). Novel high-throughput approach for purification of infectious virions. Sci. Rep. 6, 36826. 10.1038/srep36826 27827454 PMC5101806

[B21] Jindal N. Patnayak D. P. Chander Y. Ziegler A. F. Goyal S. M. (2010). Detection and molecular characterization of enteric viruses from poult enteritis syndrome in turkeys. Poult. Sci. 89, 217–226. 10.3382/ps.2009-00424 20075272 PMC7107190

[B22] Kolmogorov M. Yuan J. Lin Y. Pevzner P. A. (2019). Assembly of long, error-prone reads using repeat graphs. Nat. Biotechnol. 37, 540–546. 10.1038/s41587-019-0072-8 30936562

[B23] Koren S. Walenz B. P. Berlin K. Miller J. R. Bergman N. H. Phillippy A. M. (2017). Canu: scalable and accurate long-read assembly via adaptive k-mer weighting and repeat separation. Genome Res. 27, 722–736. 10.1101/gr.215087.116 28298431 PMC5411767

[B24] Li H. (2011). A statistical framework for SNP calling, mutation discovery, association mapping and population genetical parameter estimation from sequencing data. Bioinformatics 27, 2987–2993. 10.1093/bioinformatics/btr509 21903627 PMC3198575

[B25] Li H. (2016). Minimap and miniasm: fast mapping and *de novo* assembly for noisy long sequences. Bioinformatics 32, 2103–2110. 10.1093/bioinformatics/btw152 27153593 PMC4937194

[B26] Li H. Durbin R. (2010). Fast and accurate long-read alignment with Burrows-Wheeler transform. Bioinformatics 26, 589–595. 10.1093/bioinformatics/btp698 20080505 PMC2828108

[B27] Li H. Handsaker B. Wysoker A. Fennell T. Ruan J. Homer N. (2009). The sequence alignment/map format and SAMtools. Bioinformatics 25, 2078–2079. 10.1093/bioinformatics/btp352 19505943 PMC2723002

[B28] Liu D. Zou Z. Song S. Liu H. Gong X. Li B. (2023). Epidemiological analysis of avian reovirus in China and research on the immune protection of different genotype strains from 2019 to 2020. Vaccines (Basel) 11, 485. 10.3390/vaccines11020485 36851362 PMC9960544

[B29] Li Z. Chen Y. Mu D. Yuan J. Shi Y. Zhang H. (2011). Comparison of the two major classes of assembly algorithms: overlap–layout–consensus and de-bruijn-graph. Briefings Funct. Genomics 11, 25–37. 10.1093/bfgp/elr035 22184334

[B30] Lu H. Tang Y. Dunn P. A. Wallner-Pendleton E. A. Lin L. Knoll E. A. (2015). Isolation and molecular characterization of newly emerging avian reovirus variants and novel strains in Pennsylvania, USA, 2011-2014. Sci. Rep. 5, 14727. 10.1038/srep14727 26469681 PMC4606735

[B44] Markis M. (2022). Evaluation of pathogenicity and antigenicity of avian reoviruses and disease control through vaccination. Avian Dis. 66 (4), 435–442. 10.1637/aviandiseases-D-22-99994 36715476

[B31] Mase M. Gotou M. Inoue D. Masuda T. Watanabe S. Iseki H. (2021). Genetic analysis of avian reovirus isolated from chickens in Japan. Avian Dis. 65, 346–350. 10.1637/0005-2086-65.3.340 34427406

[B32] Moura-Alvarez J. Chacon J. V. Scanavini L. S. Nuñez L. F. Astolfi-Ferreira C. S. Jones R. C. (2013). Enteric viruses in Brazilian Turkey flocks: single and multiple virus infection frequency according to age and clinical signs of intestinal disease. Poult. Sci. 92, 945–955. 10.3382/ps.2012-02849 23472018 PMC7107160

[B33] Nour I. Alvarez-Narvaez S. Harrell T. L. Conrad S. J. Mohanty S. K. (2023). Whole genomic constellation of avian reovirus strains isolated from broilers with arthritis in North Carolina, USA. Viruses 15, 2191. 10.3390/v15112191 38005869 PMC10675200

[B34] Palomino-Tapia V. Mitevski D. Inglis T. VAN DER Meer F. Abdul-Careem M. F. (2018). Molecular characterization of emerging avian reovirus variants isolated from viral arthritis cases in Western Canada 2012-2017 based on partial sigma (σ)C gene. Virology 522, 138–146. 10.1016/j.virol.2018.06.006 30029013

[B35] Parris D. J. Kariithi H. Suarez D. L. (2022). Non-target RNA depletion strategy to improve sensitivity of next-generation sequencing for the detection of RNA viruses in poultry. J. Vet. Diagn Invest 34, 638–645. 10.1177/10406387221102430 35791437 PMC9266509

[B36] Sievers F. Higgins D. G. (2014). Clustal Omega, accurate alignment of very large numbers of sequences. Methods Mol. Biol. 1079, 105–116. 10.1007/978-1-62703-646-7_6 24170397

[B37] Spackman E. Pantin-Jackwood M. Day J. M. Sellers H. (2005). The pathogenesis of Turkey origin reoviruses in turkeys and chickens. Avian Pathol. 34, 291–296. 10.1080/03079450500178501 16147564

[B38] Tang Y. Lin L. Sebastian A. Lu H. (2016). Detection and characterization of two co-infection variant strains of avian orthoreovirus (ARV) in young layer chickens using next-generation sequencing (NGS). Sci. Rep. 6, 24519. 10.1038/srep24519 27089943 PMC4835796

[B39] Tang Y. Yu H. Y. Jiang X. N. Bao E. D. Wang D. Lu H. G. (2022). Genetic characterization of a novel pheasant-origin orthoreovirus using Next-Generation Sequencing. Plos One 17, e0277411. 10.1371/journal.pone.0277411 36409667 PMC9678273

[B40] Vigil K. Aw T. G. (2023). Comparison of *de novo* assembly using long-read shotgun metagenomic sequencing of viruses in fecal and serum samples from marine mammals. Front. Microbiol. 14, 1248323. 10.3389/fmicb.2023.1248323 37808316 PMC10556685

[B41] Vijaykrishna D. Mukerji R. Smith G. J. (2015). RNA virus reassortment: an evolutionary mechanism for host jumps and immune evasion. PLoS Pathog. 11, e1004902. 10.1371/journal.ppat.1004902 26158697 PMC4497687

[B42] Wang Y. Zhao Y. Bollas A. Wang Y. Au K. F. (2021). Nanopore sequencing technology, bioinformatics and applications. Nat. Biotechnol. 39, 1348–1365. 10.1038/s41587-021-01108-x 34750572 PMC8988251

[B43] Wick R. R. Judd L. M. Gorrie C. L. Holt K. E. (2017). Unicycler: resolving bacterial genome assemblies from short and long sequencing reads. PLoS Comput. Biol. 13, e1005595. 10.1371/journal.pcbi.1005595 28594827 PMC5481147

