# Supplementary material for: Choosing the most suitable NGS technology to combine with a standardized viral enrichment protocol for obtaining complete avian orthoreovirus genomes from metagenomic samples
Source: Front Bioinform. 2025 Feb 4;5:1498921. doi: 10.3389/fbinf.2025.1498921 (PMC11833334; doi:10.3389/fbinf.2025.1498921)
Supplement: Supplementary file 1 [file Table1.docx]

**SUPPLEMENTARY MATERIALS:**

Figure S1. In house pipeline for ARV reference-guided assemblies


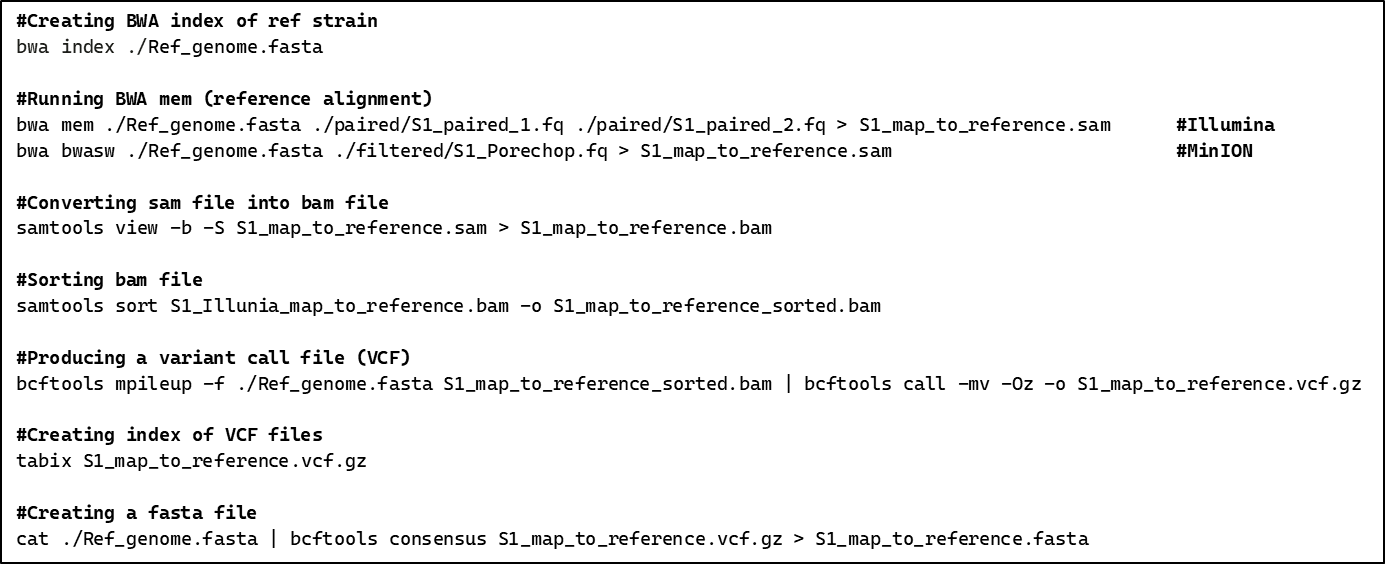


Table S2. Custom genomes used in the reference- based assemblies

Table S3. Illumina sequencing run performance

| Seq ID | Isolate ID | Raw reads | Filtered reads | Filtered ARV-mapping reads | Filtered chicken-mapping reads | |
| --- | --- | --- | --- | --- | --- | --- |
| S1 | 1197 | 225,468 | 151,575 (67%) | 91,199 (60%) | | 20,544 (14%) |
| S2 | 1148 | 135,606 | 82,524 (61%) | 79,845 (97%) | | 18 (0.02%) |
| S3 | 1110 | 17,720 | 9,860 (56%) | 8,856 (90%) | | 26 (0.3%) |
| S4 | 1143 | 126,600 | 68,456 (54%) | 66,018 (96%) | | 32 (0.05%) |
| S5 | 1072 | 179,450 | 89,302 (50%) | 88,436 (99%) | | 28 (0.03%) |
| S6 | 1064 | 99,000 | 60,045 (61%) | 58,213 (97%) | | 26 (0.05%) |
| S7 | 1087 | 119,480 | 65,588 (55%) | 64,649 (99%) | | 14 (0.02%) |
| S8 | 1088 | 32,922 | 14,984 (46%) | 14,518 (97%) | | 2 (0.01%) |

Table S4. ONT sequencing run performance

Table S5. Number of Illumina (short) and ONT (long) reads that mapped to the ARV S1133 genome or to the custom genome.

Table S6. Total number of different bp between the different assembly methods
